# Cognitive impairment and “invisible symptoms” are not associated with CCSVI in MS

**DOI:** 10.1186/1471-2377-13-97

**Published:** 2013-07-27

**Authors:** Carmela Leone, Emanuele D’Amico, Sabina Cilia, Alessandra Nicoletti, Luigi Di Pino, Francesco Patti

**Affiliations:** 1 Department GF Ingrassia, Section of Neurosciences, University of Catania, Catania, Italy; 2 Department of Cardiology, University of Catania, Catania, Italy

## Abstract

**Background:**

We investigated the association between chronic cerebrospinal venous insufficiency (CCSVI) and cognitive impairment (CI) in multiple sclerosis (MS). Moreover, we evaluated the association between CCSVI and other frequent self-reported MS symptoms.

**Methods:**

We looked at the presence of CI in incident MS patients with CCVSI in a population-based cohort of Catania, Italy. All subjects were group-matched by age, sex, disease duration and EDSS score with MS patients without CCSVI, serving as controls. CI was assessed with the Brief Repeatable Battery (BRB) and the Stroop Test (ST) and it was defined by the presence of at least three impaired tests. Fatigue and depressive symptoms were assessed with Fatigue Severity Scale (FSS) and Hamilton Depressive Rating Scale (HDRS), respectively. Bladder and sexual symptoms were assessed with the respective items of the Italian version of Guy's Neurological Disability Scale (GNDS). Quality of life was evaluated with Multiple Sclerosis Quality of Life-54 Instrument (MSQOL-54).

**Results:**

Out of 61 MS patients enrolled in the study, 27 were CCSVI positive and 34 were CCSVI negative. Of them, 43 were women (70.5%); the mean age was 43.9 ± 11.8 years; the mean disease duration was 159.7 ± 113.7 months; mean EDSS was 3.0 ± 2.6. Of them, 36 (59.0%) were classified relapsing-remitting (RR), 12 (19.7%) secondary progressive (SP), seven (11.5%) primary progressive (PP) and six (9.3%) Clinically Isolated Syndrome (CIS). Overall, CI was detected in 29/61 (47.5%) MS patients; particularly 13/27 (48.1%) in the CCSVI positive group and 16/34 (47.0%) in the CCSVI negative group. Presence of CCSVI was not significantly associated with the presence of CI (OR 1.04; 95% CI 0.37-2.87; p-value = 0.9). Not significant differences were found between the two groups regarding the other MS symptoms investigated.

**Conclusions:**

Our findings suggest a lack of association between CCSVI and CI in MS patients. Fatigue, depressive, bladder/sexual symptoms and self-reported quality of life are not associated with CCSVI.

## Background

Multiple sclerosis (MS) is an inflammatory-mediated demyelinating disease of the Central Nervous System (CNS), characterized also by axonal damage in the brain and spinal cord [1] and cortical lesions [2]; its pathogenesis is still unknown, but it is most likely caused by a complex interplay between polygenetic and environmental factors [3,4].

Recently, it was hypothesized that extra-cranial venous abnormalities, named chronic cerebrospinal venous insufficiency (CCSVI), might play a role in the pathogenesis of MS [5,6]. CCSVI has been described to interfere with venous drainage from the CNS, which contributes to the development and progression of MS; moreover, when the stenotic lesions in the internal jugular and azygos veins were treated with balloon angioplasty, rapid and often dramatic symptomatic improvement in many patients has been reported [7]. However, a number of studies have showed controversial data (ranges from 0% to 100%) about the frequency of CCSVI in MS patients so far see in review, [8]. Recently, our group found that CCSVI was present in 18.9% of MS patients [9], but one more recent study did not find any association between CCSVI and MS [10]. Although several studies have recently evaluated the effects of CCSVI endovascular treatment on subjective aspects of MS, such as fatigue, patients clinical status and their quality-of-life [7,11-14], to date no studies have investigated the possibility of an association between CCSVI and symptoms of MS.

MS determines not only motor symptoms and signs which are responsible for the so-called physical disability, but it is also responsible for a number of non-motor, self-reported symptoms and signs so-called “invisible symptoms” [15]. Among them, in recent years, much attention has been focused on bladder and sexual disorders, visual symptoms, fatigue, pain, depression and anxiety, and especially, on cognitive disorders.

Cognitive impairment (CI) is a well-recognized symptom of MS, occurring in about half of all patients; it is a disabling symptom which contributes to the poor quality of life in MS patients [16]. CI is a common finding in brain vascular diseases also; however, because of evolving definitional criteria, the precise frequency of vascular CI is difficult to assess [17]. The cerebral flow reduction, which is a key feature of brain vascular diseases, has been recently investigated in MS [18]. A widespread cerebral hypo perfusion seen in MS [18], leading to brain atrophy, could be the result of the venous outflow obstructions secondary to CCSVI [19].

We aimed to investigate whether CCSVI may be associated with CI in MS patients. Bearing in mind that CI could be a direct effect of MS or an effect of the cerebrovascular hypo perfusion, we sought to investigate the presence of a possible association between CCSVI and CI in MS patients.

## Methods

### Ethics statement

The study was approved by the two different local ethical committees (Azienda Universitaria-Ospedaliera Policlinico Vittorio Emanuele di Catania and Ethical Committe of the Azienda Sanitaria Locale 3 of Catania). Patients and controls were enrolled after they had signed the informed consent.

### Study population

We recently carried out a population-based case–control study to evaluate the possible association between MS and CCSVI [9]. Briefly, from 1 January 1975 to 31 December 2004, 367 MS patients resident in the study area had had the onset of disease [20-22]. Presence of CCSVI was evaluated in 148 MS patients randomly selected from this well defined incident-cohort and in 20 patients affected by CIS. All MS subjects enrolled in the study had fulfilled Poser criteria for Clinically Defined MS [23]. All CIS subjects enrolled had fulfilled McDonald criteria [24]. CCSVI, defined according to the Zamboni’s criteria, was found in 28 of the 148 MS patients, and in 2 of the CIS patients. Details are described elsewhere [9]. To evaluate the possible association between CI and CCSVI we enrolled in the present study, all MS and CIS patients who fulfilled the diagnostic criteria for CCSVI identified in the previous population-based case–control study [9]. Control subjects were selected among the 130 patients without CCSVI and were “frequency matched” by age (± five years), sex, mean EDSS, mean disease duration and disease course. Exclusion criteria were the presence of known vascular malformations and mental illness (history of psychiatric disorders including suicidal ideation or any episode of clinically severe depression diagnosed by DSMIV [25]), history of chronic drug or alcohol abuse prior to neuropsychological (NPS) examination, any traumatic history occurring within 3 months prior to NPS examination and pregnancy. Patients who had experienced a MS relapse within the 50 days prior to study entry were excluded from the study.

### Clinical and exposure assessment

As described elsewhere, the presence of CCSVI was defined as the presence of at least two out of the follow five parameters [5]:

I. Reflux in the IJV and/or VVs in sitting and supine posture;

II. Reflux in the DCVs;

III. High-resolution B-mode evidence of IJV stenosis;

IV. Flow not Doppler detectable in the IJVs and/or VVs;

V. Reverted postural control of the main cerebral venous outflow pathways.

ECD and TCC ultrasonographies were performed by an experienced vascular sonographer who attended a course on CCSVI at the University of Ferrara in 2011 (supervisor Dr Zamboni, who first described this modality of CCSVI assessment in MS). All enrolled patients underwent a complete physical examination, including blood pressure measurement and detailed medical history; they also underwent a CT angiography to evaluate other possible causes of cerebrovascular disease.

### NPS assessment

All neurological examinations were performed by trained and certified examining neurologist [Neurostatus, 2006; available at http://www.neurostatus.net]; for each patient the Expanded Disability Status Scale (EDSS) [26] was recorded the same day of NPS assessment, which was obtained within two weeks from Echo Color Doppler examination.

The NPS assessments included Rao’s Brief Repeatable Battery (BRB) [27,28] and the Stroop Colour-Word Task (ST) [29] for cognitive domains. All patients underwent cognitive testing for the first time and took the same form of the BRB test (Form A). The BRB incorporates tests of verbal memory acquisition and delayed recall (Selective Reminding Test, SRT) [30], visual memory acquisition and delayed recall (10/36 Spatial Recall Test, SPART) [31], attention, concentration, and speed of information processing (Paced Auditory Serial Addition Test, PASAT [32]; Symbol Digit Modalities Test, SDMT) [33], and verbal fluency on semantic stimulus (Word List Generation, WLG) [34]. All NPS tests were administered in the following order: SRT Long Term Storage (LTS, SRT Consistent Long-Term Retrieval (SRT-CLTR), SPART, SDMT, PASAT 3′, PASAT 2′, SRT-Delayed (SRT-D), SPART-Delayed (SPART-D), WLG-A and ST. All the patients were examined by the same neuropsychologist in order to uniform criteria of administration, data recording and scoring procedures. In order to guarantee the blinding of the neuropsychologist, we instructed subjects not to reveal their CCSVI status during NPS examination. Failure on a test was defined using the available normative data for the Italian population, considering the fifth percentile of the Italian population performance on each evaluation as the cut-off point for calculating the number of failed tests of the BRB; for the ST we considered the ninety-fifth percentile [27]. Cut-off points were applied to the scores adjusted by age and education [27].

Patient intelligence quotient (IQ) was determined before the NPS assessment by administering the Brief Intelligence Test (BIT) [35]. The BIT is based on the correlation between general intelligence and reading ability. The test comprises a reading skill test of 54 words with either regular or irregular emphasis (accent) [35,36].

Quality of life (QoL) was assessed by the MSQoL-54 questionnaire [37], depressive symptoms by the Hamilton Depression Rating Scale (HDRS) [38], fatigue by the Fatigue Severity Scale (FSS) [39], bladder and sexual symptoms by the respective items of the Italian version of GNDS [40]. These tests were self-administered one-three days before NPS testing.

### Statistical analysis

Data were analyzed using STATA 10.0 software packages [41]. Data were double entered into the database. Data cleaning was also performed before the data analysis considering both range and consistence checks. Quantitative variables were described using mean and standard deviation (mean ± SD). Means and proportions were assessed by t-test and Chi-square test respectively. In case of not a normal distribution appropriate non-parametric tests were performed. Unconditional logistic regression analysis was performed and for each study variable, we calculated OR, 95% CI, and p value (two-tailed test, p = 0.05). Parameters associated with the outcome at the univariate analysis with a threshold of p = 0.10 were included in the model. The model was manually constructed using the Likelihood Ratio Test (LRT) to compare the log-likelihood of the model with and without a specific variable.

Multiple linear regression analysis was used to test whether demographic and clinical characteristics including CCSVI, can predict the cognitive outcome. The number of failed subtests was selected as the variable representing the extent of cognitive decline.

Whenever variables were dichotomized or polychotomized, the cut-offs were derived from the pooled distribution of cases and control subjects (e.g., using the median, tertiles, or quartiles).

## Results

Out of the 30 patients (28 MS and 2 CIS) CCSVI positive identified in the previous population-based case–control study, 27 (25 MS and two CIS) were enrolled in the study. Out of the 138 MS and CIS patients without CCSVI, identified in the previous study, using a frequency matching, 34 similar in terms of age (± 5 years), sex, mean EDSS, mean disease duration and disease course were enrolled in the study as control group.

Demographic and clinical characteristics of patients with and without CCSVI are reported in Table 1.

**Table 1:** Baseline demographic and clinical characteristics of study sample

	**CCSVI-**	**CCSVI+**	**p-value**
**N° (%)**	34	27	0.3
**W/M (%)**	24 (70.5)	19 (70.3)	1.0
**Age (mean ± SD)**	42.3 ± 12.3	46.1 ± 11.1	0.3
**EDSS (mean ± SD)**	3.1 ± 2.8	3.0 ± 2.3	1.0
**Disease Duration (years; mean ± SD)**	13 ± 9.9	13.7 ± 8.8	0.6
**Mean Education (years; mean ± SD)**	11.9 ± 3.9	12.1 ± 4.6	0.7
**RR (%)**	19 (55.9)	17 (63.0)	0.5
**SP (%)**	9 (26.5)	3 (11.1)	0.2
**PP (%)**	2 (5.9)	5 (18.5)	0.2
**CIS (%)**	4 (11.8)	2 (7.4)	0.7

Data are given as means ± standard deviations.

***W*** women.

***M*** men.

***RR*** relapsing-remitting.

***SP*** secondary progressive.

***PP*** primary progressive.

***CIS*** clinically isolated syndrome.

The most common venous abnormalities found in our patients are detailed in Table 2.

**Table 2:** Distribution of venous hemodynamic criteria among CCSVI positive patients

	**Number of CCSVI positive patients (%)**
**Criterion III**	19 (28.8%)
**Criterion II**	16 (24.2%)
**Criterion I**	15 (22.7%)
**Criterion V**	15 (22.7%)
**Criterion IV**	1 (1.5%)

**Criterion I =** Reflux in the IJVs and/or VVs in sitting and supine posture.

**Criterion II =** Reflux in the DCVs.

**Criterion III =** High-resolution B-mode evidence of proximal IJV stenoses.

**Criterion IV =** Flow not Doppler detectable in the IJVs and/or VVs.

**Criterion V =** Reverted postural control of the main cerebral venous outflow Pathway (Δ CSA).

***N*** number.

***IJVs*** internal jugular veins.

***VVs*** vertebral veins.

***DCVs*** deep cerebral veins.

CI was present in 13/27 CCSVI positive MS-patients and in 16/34 CCSVI negative MS-patients (48.1% and 47.0%, respectively). At univariate analysis presence of CCSVI was not associated with presence of CI (OR 1.04; 95% CI 0.37-2.87; p-value = 0.9). A close association (OR 0.90; 95% CI 0.23-3.53; p-value = 0.8) was found performing a multivariate analysis and adjusting by age sex and EDSS considered as a priori confounders and included in the model regardless the p-value threshold.

According to multiple linear regression analysis only increasing age (p-value 0.01), followed by higher EDSS (p-value 0.08), proved to be positively correlated with the severity of cognitive dysfunction defined as the number of test failed as shown in Table 3.

**Table 3:** Multiple linear regression analysis: predictors of cognitive dysfunction

**Outcome**	**Predictors**	**Coefficient**	**p-value**
**Failed subtests**	CCSVI	.02	0.9
	Sex	-.45	0.4
	Age	.06	0.01
	EDSS	.20	0.08

**Figure 1:**
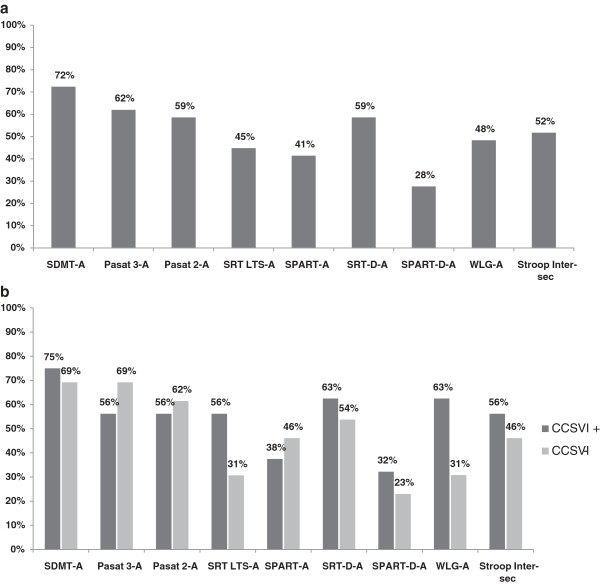
(a) Frequency distribution of impaired NPS tests among cognitive impaired MS pts. (b) Frequency distribution of impaired NPS tests among CCSVI + and CCSVI- MS pts.

The most frequently impaired tests found in CI patients were SDMT, 3′ PASAT and 2′ PASAT, SRT-D and ST (see Figure 1a). For the cognitive profile, no significant difference was found between the two groups (see Figure 1b). Regarding the self-reported measures of fatigue, depressive symptoms, bladder/sexual disturbances and quality of life not significant differences between CCSVI positive and CCSVI negative patients were found (see Table 4).

**Table 4:** Self-report MS symptoms measures at the time of evaluation

	**CCSVI-**	**CCSVI+**	**p-value**
**FSS (mean ± SD)**	3.7 ± 1.7	3.0 ± 1.8	0.7
**HDRS (mean ± SD)**	5.0 ± 3.7	6.7 ± 4.6	0.7
**Bladder symptoms GNDS (mean ± SD)**	1 ± 1.4	0.8 ± 1.0	0.7
**Sexual symptoms GNDS (mean ± SD)**	1 ± 1.8	0.96 ± 1.4	0.6
**MSQOL-54-PHCS (mean ± SD)**	65.3 ± 21.4	70.3 ± 17.2	0.2
**MSQOL-54-MHCS (mean ± SD)**	64.2 ± 20.4	67.5 ± 19.6	0.2

Data are given as means ± standard deviations.

***FSS*** Fatigue Severity Scale.

***HDRS*** Hamilton depression Rating Scale.

***GNDS*** Guy’s Neurological Disability Scale.

***MSQOL-54*** Multiple Sclerosis Quality of Life-54.

***PHCS*** Physical Health Composite Score.

***MHCS*** Mental Health Composite Score.

## Discussion

Our study, performed in a well-defined MS population, showed that CI was not associated with CCSVI. We found about the same prevalence of CI between CCSVI-positive (48.1%) and CCSVI-negative patients (47.0%). Moreover, a similar prevalence of other frequent reported MS-symptoms, such as fatigue, depression and bladder/sexual disturbances, was found between the two groups. These findings suggest that CCSVI is not correlated to the cognitive status and other MS symptoms investigated, at least in our well-selected MS population.

Our study is the first evaluating the possible association between CCSVI and one of the most disabling MS symptoms such as CI. In the few years, a number of studies were carried out to evaluate the possible association between CCSVI and MS; most of them reported higher frequency of CCSVI among MS patients and suggested a possible pathogenic role of CCSVI in determining specific symptoms and signs [5,8]. Although our recent population-based study [9] demonstrated that CCSVI was associated with MS, especially with progressive forms of the disease, there is no agreement about the exact frequency of CCSVI in MS, its putative pathogenic role and its contribution, if any, on MS symptoms [5,8,42-45]. CI is one of the most common and disabling symptom of MS, occurring in about half of all MS patients. Literature data show that the most common cognitive deficits in MS patients are memory and speed of information processing, concentration and executive functions [16,46]. However, the mechanisms underlying CI in MS has not been fully elucidated. Several cross sectional studies demonstrated that the pathogenesis of CI in MS patients might not depend just on the extent and severity of the pathological process in the brain lesions, but also on the pathological changes affecting the normal appearing brain tissue [47-49]. Considering that, we could take into account the possible vascular hypothesis as a possible contribute to the presence of a spread or regional brain atrophy, which is one of the strongest factor correlated with the CI in MS [50-52]. Impaired cerebral perfusion seems to be related to CI [18] and a widespread cerebral hypo perfusion in MS, described as a potential cause of brain atrophy [50-52], could be the result of the venous outflow obstructions as seen in the CCSVI [19]. Although there is no strong evidence suggesting that CCSVI is a cause of MS, there is some evidence that a slower cerebral venous flow in patients with MS might be secondary to the reduced cerebral blood flow [19].

Our results do not support the hypothesis that CCSVI might be responsible for the appearance or worsening of CI in MS.

This study represents the first population-based case control-study looking at the possible association between CCSVI and CI in MS.

The population-based design represents the main strength of our study reducing the risk of a possible selection bias. Nevertheless such design has led to a small study size allowing us to enrol just a limited number of cases and controls. Consequently the lack of association between CCSVI and CI in MS patients could be due to a lack of power (type II error). Furthermore the retrospective nature of the study did not allow us to establish the exact sequence of the events. In order to exclude a number of conditions which might have influenced the NPS performance, we evaluated subjective fatigue and depressive symptoms, excluding that these symptoms could be correlated with CCSVI.

CI was detected by a single trained Neuropsychologist in order to avoid any inter-rater disagreement.

Although the role of CCSVI in MS pathology is still unknown and the previous evidence are controversial so far, further prospective studies are needed to withdraw the hypothesis that CCSVI might play a role in determining CI or other invisible symptoms in MS.

## Conclusions

Our findings suggest a lack of association between the presence of CCSVI with CI and others “invisible symptoms” in patients with MS.

## Abbreviations

BRB: Rao’s brief repeatable battery; CCSVI: Chronic cerebrospinal venous insufficiency; CI: Cognitive impairment; CIS: Clinically isolated syndrome; EDSS: Expanded disability status scale; FSS: Fatigue severity scale; GNDS: Guy’s neurological disability scale; HDRS: Hamilton depressive rating scale; MR: Magnetic resonance; MS: Multiple sclerosis; NPS: Neuropsychological; OR: Odds ratio; RR: Relapsing-remitting; SP: Secondary progressive; PP: Primary progressive.

## Competing interests

### Financial competing interests

PF has received honoraria for speaking activities by Bayer Schering, Biogen Idec, Merck Serono, Novartis and Sanofi Aventis; he also served as advisory board member the following companies: Bayer Schering, Biogen Idec, Merck Serono, Novartis; he was also funded by Pfeizer and FISM for epidemiological studies; finally he received grant for congress participation from Bayer Schering, Biogen Idec, Merck Serono, Novartis, Sanofi Aventis and TEVA. LC received partial grant for congress participation from Bayer Schering and Biogen Idec. DAE received grants for congress participation from Sanofi Aventis. CS declares that she has not competing interests. NA declares that she has not competing interests. DPL declares that he has not competing interests.

### Non financial competing interests

This study was not sponsored even if RELOAD ONLUS supported the study, providing the tilt table used in the study.

## Authors’ contributions

LC participated in the design of the study and in manuscript preparation. DAE performed neurological examination of patients. CS performed neuropsychological examination of patients. NA participated in the study design, manuscript preparation and performed the statistical analyses. DPL carried out the ECD and TCC evaluations. PF conceived the study and participated in its design and coordination. All authors read and approved the final manuscript.

## Pre-publication history

The pre-publication history for this paper can be accessed here:

http://www.biomedcentral.com/1471-2377/13/97/prepub
